# Real-World Observational Clinical Study Assessing the Effectiveness and Safety of Vonoprazan-Based Dual Therapy Versus Proton Pump Inhibitor-Based Triple Therapy for *Helicobacter pylori* Eradication

**DOI:** 10.14740/gr2148

**Published:** 2026-06-16

**Authors:** Sanjay Bandyopadhyay, Gajanan Panchal, Maneesha Khalse, Kamlesh Patel

**Affiliations:** a Department of Gastroenterology, Kolkata Gastro Care, Kolkata, W.B., India; b Department of Medical Affairs, Lupin Ltd., Mumbai, Maharashtra, India

**Keywords:** *Helicobacter pylor*i, Vonoprazan, Proton pump inhibitors, Dual therapy, Triple therapy, Eradication rate

## Abstract

**Background:**

*Helicobacter pylori* (*H. pylori*) is a gram-negative bacterium linked to various gastrointestinal disorders. Our study evaluates the comparative effectiveness, safety, and cost-efficiency of vonoprazan–amoxicillin (VA) dual therapy as an alternative to traditional proton pump inhibitor (PPI)-based triple therapy for eradicating *H. pylori*.

**Methods:**

This real-world, observational, single-center clinical study used medical records of 94 adult patients diagnosed with *H. pylori* infection in India between July 2024 to March 2025. The patients received 14 days of VA dual therapy or PPI–amoxicillin–clarithromycin (PAC) triple therapy. The primary outcome included *H. pylori* eradication using a repeat rapid urease test, and the secondary outcomes included the need for rescue therapy, time for symptom resolution, incidence of adverse drug reactions, and cost-comparison of the treatment regimens.

**Results:**

Eradication rates were numerically higher with VA than PAC (93.33% vs. 85.71%), although the difference was not statistically significant. As second-line therapy, eradication was achieved in 100% of PAC-to-VA switchers and 33.3% of VA-to-PAC switchers. Symptom resolution was significantly earlier with VA compared to PAC (median, 3 vs. 5 days; P < 0.001). Adverse events were fewer with VA (13.33%) than PAC (28.57%), with taste disturbances more frequent in the PAC group. VA also showed a significantly lower median treatment cost (Indian rupee (INR) 1,020 (US dollar (USD) 12.29) vs. INR 3,380 (USD 40.72); P <0.001) and a better cost-comparison ratio (10.67 vs. 39.43).

**Conclusions:**

VA showed numerically higher eradication rates, faster symptom relief, lower costs, and better tolerability than PAC in the Indian setting, supporting its potential as a practical alternative first-line regimen, particularly in settings where clarithromycin resistance is prevalent or suspected.

## Introduction

*Helicobacter pylori* (*H. pylori*) is a gram-negative, microaerophilic bacterium that colonizes the human gastric mucosa and is associated with a spectrum of gastrointestinal disorders, including chronic gastritis, peptic ulcer disease, mucosa-associated lymphoid tissue (MALT) lymphoma, and gastric cancer [1–3]. The global prevalence of *H. pylori* has shown a declining trend over recent decades, from 58.2% in the 1980s to 43.1% by 2022, with significant regional variability. However, in developing countries like India, infection remains endemic, with estimates suggesting that up to 80% of the population may be infected by early adulthood [4]. In rural areas, this infection rate can be even higher, exceeding 80% of the population. Peptic ulcer disease, especially duodenal ulcers, is the most common health issue linked to *H. pylori* in India [5].

According to the Indian Consensus statement, the current first-line therapy for *H. pylori* includes a proton pump inhibitor (PPI), amoxicillin, and clarithromycin, administered for 14 days. However, increasing antibiotic resistance, particularly to clarithromycin, metronidazole, and fluoroquinolones, has led to reduced efficacy of standard regimens. Alternate therapies such as bismuth-based quadruple therapies and salvage regimens are emerging, but these are often limited by complexity, poor tolerability, and poor adherence. Current recommended treatments for *H. pylori* infection generally involve a combination of antibiotics together with an acid suppressant, such as a PPI. However, treatment success rates with current guideline-recommended PPI-based regimens remain suboptimal [6].

Vonoprazan, a potassium-competitive acid blocker (P-CAB), was approved for clinical use in India in 2024 for the treatment of acid-related disorders, including *H. pylori* eradication therapy [7]. It provides more potent and sustained acid suppression compared to conventional PPIs [8, 9]. Multiple phase 3 clinical trials, including the international PHALCON-HP study, have demonstrated that vonoprazan-based dual therapy (vonoprazan + amoxicillin) achieves comparable or higher eradication rates with better tolerability, including in settings where clarithromycin resistance may reduce the effectiveness of PPI–amoxicillin–clarithromycin (PAC) therapy of *H. pylori* [10]. Furthermore, pooled analyses of phase 3 studies conducted in East Asia reported eradication rates exceeding 90% with vonoprazan-based regimens, outperforming standard PPI-based therapies [11]. Clinical studies in East Asia [12] have demonstrated that vonoprazan–amoxicillin (VA) therapy achieves comparable or superior eradication rates with fewer antibiotics and better tolerability [7]. Although promising results have been reported from the studies done in Japan and Korea [12], these regimens have not been sufficiently studied in the Indian population, where region-specific evidence is crucial for informing treatment decisions.

A lack of real-world evidence from the Indian population prompted this study, which evaluates the comparative effectiveness, safety, and cost comparisons of VA-dual therapy versus PPI-based triple therapy in routine Indian clinical settings.

## Materials and Methods

### Study design

This was a retrospective, real-world, observational, comparative clinical study conducted at a single center. The study reviewed medical records of patients treated over a 9-month period, from July 2024 to March 2025. Data were extracted from hospital records of adult patients (aged ≥ 18 years) who were diagnosed with *H. pylori* infection based on a positive rapid urease test (RUT) performed on endoscopic biopsy specimens. In addition, patients had any of the following: (1) dyspepsia for more than 6 months; (2) endoscopically documented gastric or duodenal ulcer; or (3) chronic *H. pylori*-related gastritis on gastric mucosal biopsy specimen (as per Updated Sydney protocol) or chronic duodenitis [13].

The study protocol was reviewed and approved by the Human Research Ethics Committee, (Reference No. HREC-AARC/63) of the concerned institution, with a waiver of informed consent due to the retrospective nature of data collection and the use of anonymized patient records. This study was conducted in accordance with the ethical standards of the Institutional Ethics Committee and in compliance with the principles of the Declaration of Helsinki.

### Eligibility criteria

Records of patients aged 18 years and above, who had a documented diagnosis of *H. pylori* infection confirmed by a positive RUT from endoscopic biopsy samples, were included. Only those with complete data on treatment regimens and follow-up outcomes were considered eligible. Data were extracted from hospital records of adult patients’ therapy, nonspecific biopsy findings, known hypersensitivity to study medications (vonoprazan, amoxicillin, clarithromycin, or PPIs), or significant hepatic or renal impairment (alanine aminotransferase (ALT)/aspartate aminotransferase (AST) > 3 × upper limit of normal (ULN) or creatinine clearance < 30 mL/min). Records indicating recent use (within 2 weeks) of antibiotics, PPIs, or bismuth compounds were excluded to minimize confounding. Additional exclusions were documented use of medications interacting with the study drugs (e.g., colchicine, methotrexate), history of QT interval prolongation, or concurrent QT-prolonging drugs.

Women who were pregnant or lactating at the time of treatment were excluded, as were individuals with immunocompromised conditions (e.g., human immunodeficiency virus (HIV) infection with CD4 < 200 cells/mm^3^ or immunosuppressive therapy). Patients with serious comorbid medical or psychiatric illnesses that could influence outcomes were also excluded based on physician documentation.

### Treatment regimens

The VA group received vonoprazan 20 mg twice daily (BID) and amoxicillin 1 g three times daily (TID) for 14 days. Patients in the PAC group received a PPI BID, amoxicillin 1 g BID, and clarithromycin 500 mg BID for 14 days. The PPI used in the PAC regimen varied according to routine clinical prescription and was recorded from medical records. Treatment regimens were documented in patient records at the time of prescription and follow-up. Those who did not respond to therapy were switched from PAC to VA and VA to PAC, and those who did not respond to the above two regimens were escalated to bismuth quadruple therapy. These patients received a PPI BID, bismuth 120 mg four times daily (QID), tetracycline 500 mg QID, and metronidazole 500 mg TID. Treatment allocation was based on routine physician clinical judgment, drug availability, patient affordability, and individual patient-related factors, rather than randomization.

### Outcome assessment

The primary outcome was *H. pylori* eradication, confirmed by a repeat RUT conducted 4 weeks after completing therapy. Although urea breath testing or stool antigen testing are commonly preferred noninvasive methods for confirming eradication, repeat RUT was used in this retrospective real-world study because it was the documented follow-up test available in routine clinical records. Follow-up biopsies were obtained using a standard sampling approach, and patients were advised to avoid antibiotics, bismuth compounds, and PPIs before repeat testing to reduce the possibility of false-negative results. As documented in hospital records, follow-up endoscopic biopsies (two from the antrum and one from the corpus) were obtained and tested using a standard commercial RUT kit. A positive test was defined as a color change from yellow to pink within 24 h. Patients had been advised to avoid antibiotics and PPIs for ≥ 2 weeks prior to follow-up endoscopy to reduce the risk of false-negative results [14, 15].

Secondary outcomes included the incidence, type of adverse drug reactions (ADRs), the need for rescue therapy due to treatment failure, time for symptom resolution, and cost-comparisons of the treatment regimens. ADRs were graded according to the National Cancer Institute Common Terminology Criteria for Adverse Events version 5.0 (2017) based on available clinical documentation [16]. The time to symptom resolution was evaluated as time to event for each patient, summarized as median (interquartile range (IQR)), and the results were plotted as Kaplan–Meier curves. Symptom resolution was defined as the first day on which all the presenting symptoms were absent or mild for at least 48 h. Formal adherence data were not consistently available in the medical records; therefore, treatment adherence could not be systematically evaluated in this retrospective study.

The cost effectiveness was assessed using patient-level direct medical costs obtained from the hospital’s electronic medical records. The economic analysis was conducted from a patient/direct medical cost perspective and should be interpreted as a basic cost comparison with a simple cost-per-eradication estimate, rather than a full cost-effectiveness analysis. Direct medical costs were extracted from hospital records and included the 14-day drug regimen, rescue therapy when required, medications used for ADR management, and additional clinic visits. Costs related to diagnostic endoscopy and repeat RUT were excluded equally from both groups, as these formed part of the diagnostic and outcome-assessment pathway rather than treatment-specific costs. Because cost data were skewed, median and IQR values were emphasized. Total cost included the cost for the 14-day drug regimen, rescue therapies (when required), medications used to manage ADRs, and any additional clinic visits. Costs were recorded and calculated in Indian rupee (INR) and converted to US dollar (USD) for descriptive comparison. Cost-comparison was evaluated using the cost difference (ΔC = Cost_PAC_-Cost_VA_), the cost ratio between regimens (cost ratio = median Cost_PAC_/median Cost_VA_), and the cost-effectiveness ratio (CER) (CER = median total cost/eradication rate). Antimicrobial susceptibility testing was not performed, and susceptibility data were not available in the medical records.

### Bias

Potential sources of bias included selection bias due to the retrospective inclusion of patients with complete treatment and follow-up records, confounding by indication because treatment allocation was based on routine clinical decision-making rather than randomization, and information bias related to documentation quality in medical records. Missing data were handled by complete-case analysis for the relevant variables. The single-center design and small sample size may further limit external validity. Missing data were handled by complete-case analysis for the relevant variables.

### Study size

No formal sample-size calculation was performed. The study included all eligible adult patients with documented *H. pylori* infection, treatment regimen, and follow-up outcome data available during the study period from July 2024 to March 2025. Therefore, the sample size was determined by the number of eligible records rather than by a priori power estimation.

### Statistical analysis

Data were entered into Microsoft Excel and analyzed using STATA version 17.0 (StataCorp., College Station, TX, USA). Continuous variables were summarized as mean (standard deviation (SD)), and categorical variables as frequencies and percentages. Between-group comparisons were done using Student’s *t*-test for continuous variables, Fisher’s exact test for categorical variables, and the Mann–Whitney U test for variables that were not normally distributed. The Cox proportional hazards model was considered exploratory. If the proportional hazards assumption was violated, interpretation was based primarily on Kaplan–Meier curves, log-rank test results, and median time to symptom resolution rather than the hazard ratio (HR). For skewed continuous variables, between-group differences were assessed using the Mann–Whitney U test, and results were reported as median (IQR); no median difference with 95% confidence interval (CI) was used for primary interpretation.

Kaplan–Meier survival analysis was used to evaluate time to complete symptom resolution, treated as a time-to-event outcome. Differences between treatment groups were assessed using the log-rank test. A Cox proportional hazards regression model was fitted to estimate HR with 95% CIs. The proportional hazards assumption was assessed using Schoenfeld residuals.

All analyses were two-sided, and a P value < 0.05 was considered statistically significant.

## Results

### Patient demographics and baseline characteristics

**Figure 1:**
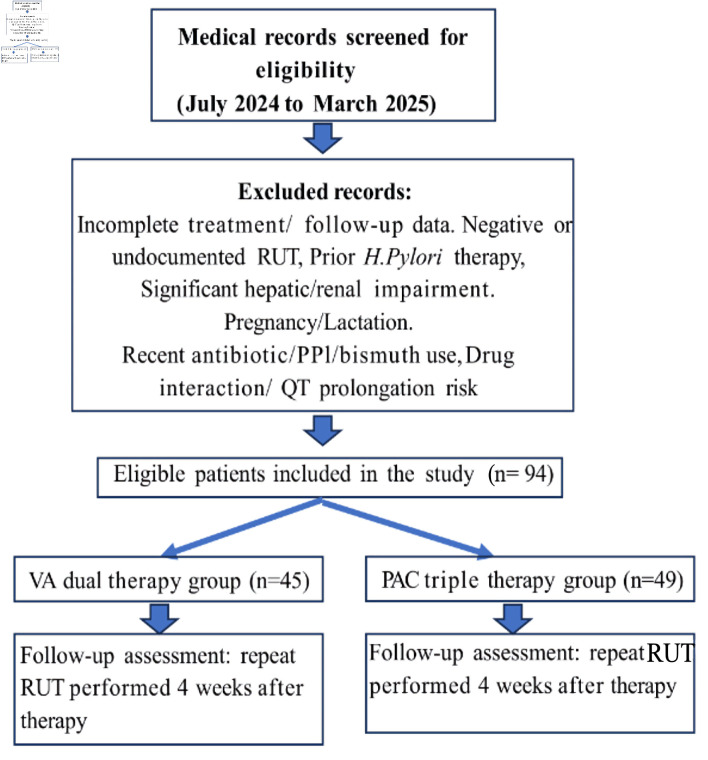
Flow diagram of patient selection and inclusion. *H. pylori*: *Helicobacter pylori*; VA: vonoprazan–amoxicillin; PAC: proton pump inhibitor–amoxicillin–clarithromycin; PPI: proton pump inhibitor; RUT: rapid urease test.

A total of 94 patients were included in the study, with 49 receiving the PAC therapy regimen and 45 receiving VA therapy (Fig. 1). The mean age of participants was 38.96 (13.18) years in the VA group and 43.49 (11.34) years in the PAC group. The males constituted 55.55% of the VA group and 42.86% of the PAC group, representing equal distribution of males and females. Regarding the baseline diagnosis, the distribution of clinical conditions was comparable between the two groups (P = 0.763). The proportions of patients diagnosed with chronic gastritis were similar in both groups (VA: 53.33% vs. PAC: 59.18%). Likewise, there were no significant differences in the prevalence of other diagnoses like duodenal ulcer (17.78% vs. 10.20%), gastric ulcer (8.89% vs. 10.20%), and chronic duodenitis (20.00% vs. 20.41%). No statistically significant differences were observed between the VA and PAC groups with respect to age, sex, or diagnostic category, indicating that the two groups were well balanced before treatment initiation. Median total treatment cost and time to complete symptom resolution were significantly lower in the VA group than in the PAC group, as shown in Table 1. Moreover, the number of adverse events (AEs) was high with the PAC treatment regimen (Table 1).

**Table 1:** Patient Demographics and Baseline Characteristics

Characteristic	VA group (n = 45)	PAC group (n = 49)	P value
Age, mean (SD)	38.96 (13.18)	43.49 (11.34)	0.078^a^
Sex			0.306^b^
Male, n (%)	25 (55.55%)	21 (42.86%)	
Female, n (%)	20 (44.45%)	28 (57.14%)	
Follow-up after first RUT positive date (number of days), mean (SD)	49.41^c^ (13.16)	70.94^c^ (75.23)	0.057^a^
Diagnosis, n (%)			0.763^b^
Chronic duodenitis	9 (20.00%)	10 (20.41%)	
Chronic gastritis	24 (53.33%)	29 (59.18%)	
Duodenal ulcer	8 (17.78%)	5 (10.20%)	
Gastric ulcer	4 (8.89%)	5 (10.20%)	
RUT result following therapy, n (%)			0.162^b^
Negative	42 (93.33%)	42 (85.71%)	
Positive	3 (6.67%)	7 (14.29%)	
Total cost of therapy (in rupees), median (IQR)	1,020 (990–1,820)	3,380 (2,980–4,280)	< 0.001^a^
Time to complete symptom resolution (days), median (IQR)	3 (3–5)	5 (5–7)	< 0.001^a^
Adverse effects, number of occurrences			–
Abdominal discomfort	0	1	–
Diarrhea	4	3	–
Headache	1	1	–
Nausea	1	6	–
Taste disturbance	1	7	–
Vomiting	0	1	–
Skin rash	1	1	–
None	39	35	–

^a^Welch’s *t*-test for normally distributed continuous variables and Mann–Whitney U test for skewed continuous variables. ^b^Pearson’s Chi-square test/Fisher’s exact test as appropriate. ^c^One observation was missing. SD: standard deviation; IQR: interquartile range; PAC: proton pump inhibitor–amoxicillin–clarithromycin; RUT: rapid urease test; VA: vonoprazan–amoxicillin.

### Effectiveness

Following first-line therapy, eradication was achieved in 93.33% of patients in the VA group compared to 85.71% in the PAC group (Table 2). However, the difference was not statistically significant (P = 0.162). Seven patients in the PAC group who failed first-line therapy were switched to VA therapy, and all achieved eradication (7/7 (100%)). Three patients in the VA group who failed first-line therapy were switched to PAC therapy, and one achieved eradication (1/3 (33.33%)). The remaining two patients achieved eradication after third-line bismuth quadruple therapy (2/2 (100%)) (Table 2).

**Table 2:** Eradication Efficacy

Outcome	VA group (n = 45)	PAC group (n = 49)	P value
First-line therapy, n (%)	42/45 (93.33%)	42/49 (85.71%)	0.162^a^
Second-line therapy, n (%)	1/3 (33.33%) VA→PAC switchers	7/7 (100%) PAC→VA switchers	0.002^a^
Third-line therapy, n (%)	2/2 (100%) after failed rescue therapy	–	–
Subgroup analysis			
Subgroup diagnosis	VA	PAC	P value
Chronic duodenitis + gastritis n (%)	33 (73.33%)	39 (79.59%)	0.63^a^
Duodenal ulcer + gastric ulcer n (%)	12 (26.67%)	10 (20.41%)	0.63 ^a^

^a^Fisher’s exact test. PAC: proton pump inhibitor–amoxicillin–clarithromycin; VA: vonoprazan–amoxicillin.

For patients diagnosed with chronic duodenitis or chronic gastritis, the eradication rates were similar between the two groups. The distribution of patients with duodenal or gastric ulcers differed numerically between groups; however, no statistically significant subgroup difference was observed after correction; this subgroup finding should be interpreted with caution given the small number of patients (Table 2).

### Symptom resolution time

**Figure 2:**
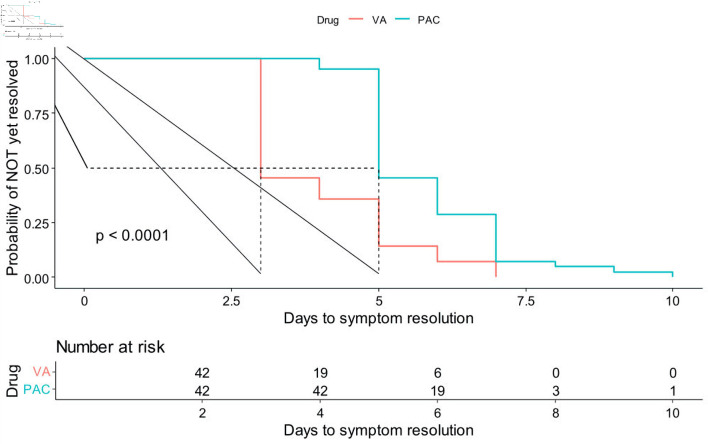
Kaplan–Meier curve comparing PAC and VA regimens (time to symptom resolution). PAC: proton pump inhibitor–amoxicillin–clarithromycin; VA: vonoprazan–amoxicillin.

**Figure 3:**
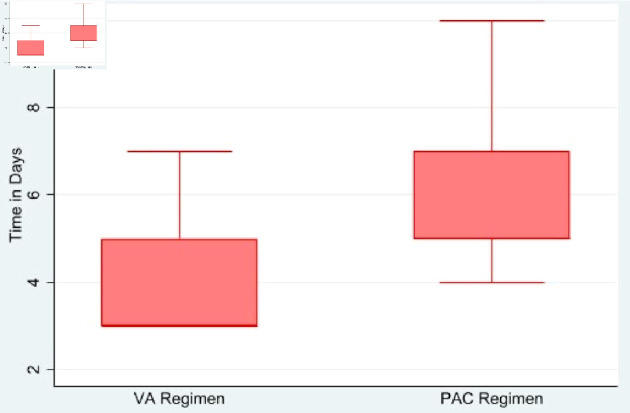
Time to symptom resolution in patients treated with VA and PAC regimens. PAC: proton pump inhibitor–amoxicillin–clarithromycin; VA: vonoprazan–amoxicillin.

Time to symptom resolution was compared for patients who responded to the first-line therapy. Kaplan–Meier analysis demonstrated a significantly faster time to complete symptom resolution in the VA group compared with the PAC group (log-rank P < 0.001) (Fig. 2). Time to complete symptom resolution was significantly shorter in the VA group (n = 42) with a median time of 3 days (IQR: 3–5, minimum: 3, maximum: 7), than in the PAC group (n = 42), which required a median of 5 days (IQR: 5–7, minimum: 4, maximum:10; P < 0.001) (Fig. 3). A significant difference was observed in the time to symptom resolution between the PAC and VA groups (P < 0.001). Because the proportional hazards assumption was violated, the Cox model estimate was interpreted cautiously. The primary interpretation was therefore based on the Kaplan–Meier curve, log-rank test, and median time to symptom resolution, which consistently indicated earlier symptom resolution in the VA group. Cox proportional hazard model analysis revealed that patients in the PAC regimen had a 60% lower rate of symptom resolution at any given time compared to the VA regimen (HR: 0.36; 95% CI: 0.23–0.56; P < 0.001). However, the Schoenfeld residual plot revealed that the proportional hazard assumption was violated (P = 0.002), indicating that the relative hazard between groups changes over time. This suggests that the relative difference in symptom resolution between the two treatment groups was not constant throughout the follow-up period and may have varied over time.

### Safety

AEs were more commonly reported in the PAC group (28.57%) compared to the VA group (13.33%), although the difference was not statistically significant (P = 0.07). Common side effects included diarrhea, nausea, and taste disturbances, with a higher frequency of taste disturbance in the PAC group vs. the VA group (12.24% vs. 2.22%). One patient in the VA group experienced three AEs, whereas none were observed in the PAC group (Table 3).

**Table 3:** Adverse Events

Adverse events, n (%)	VA group (n = 45)	PAC group (n = 49)	P value
Any event	6 (13.33%)	14 (28.57%)	0.07^a^
Two events	1 (2.22%)	5 (10.20%)	0.11^a^
Three events	1 (2.22%)	0	–
Headache	1 (2.22%)	1 (2.04%)	1.00^a^
Diarrhea	4 (8.89%)	3 (6.12%)	0.70^a^
Nausea	1 (2.22%)	4 (8.16%)	0.36^a^
Taste disturbance	1 (2.22%)	6 (12.24%)	0.11^a^
Abdominal discomfort	0	1 (2.04%)	–
Vomiting	0	1 (2.04%)	–
Skin rash	1 (2.22%)	1 (2.04%)	1.00^a^

^a^Fisher’s exact test. PAC: proton pump inhibitor–amoxicillin–clarithromycin; VA: vonoprazan–amoxicillin.

### Cost effectiveness

**Figure 4:**
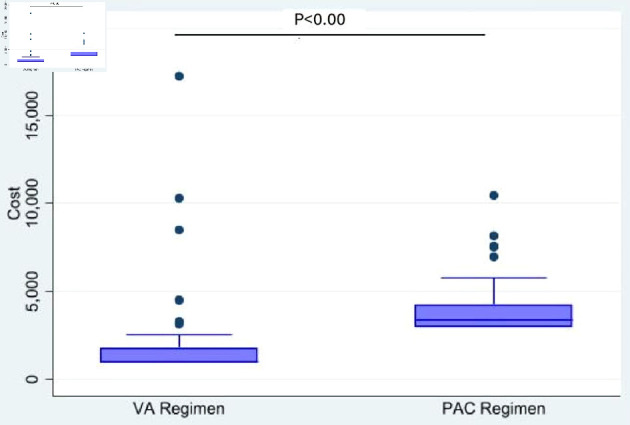
Median cost of VA and PAC regimens. PAC: proton pump inhibitor–amoxicillin–clarithromycin; VA: vonoprazan–amoxicillin.

The median total cost was significantly lower in the VA group (INR 1,020 (IQR: 990–1,820, minimum: 990, maximum: 17,270)) compared with that in the PAC group (INR 3,380 (IQR: 2,980–4,280, minimum: 2,980, maximum: 10,470); P < 0.001) (Fig. 4). This was equivalent to USD 12.29 for VA regimen and USD 40.72 for PAC regimen. The difference in the median cost between the two groups was INR 2,360 (USD 28.43), and the cost ratio of PAC versus VA group was about 3.31. Likewise, the CER of VA group was considerably lower (10.67) compared to the PAC group (39.43).

## Discussion

This real-world, observational study conducted in a tertiary care setting in India demonstrated that VA therapy showed numerically higher eradication rates, although the primary endpoint difference was not statistically significant compared to traditional PPI-based triple therapy (PAC) for *H. pylori* eradication. The VA regimen was also more cost-effective than the PAC regimen.

Specifically, the VA regimen achieved a higher eradication rate of 93.33%, compared to 85.71% with the PAC regimen. Although the difference was not statistically significant, superiority for eradication cannot be concluded from the primary endpoint. However, eradication rates were numerically higher with VA, the difference was not statistically significant (P = 0.162). Therefore, the findings should be interpreted as a trend favoring VA rather than evidence of proven superiority. The results are of clinical interest due to the lower incidence of treatment-emergent AEs in the VA group (13.33%) compared to the PAC group (28.57%). The common AEs, such as nausea and taste disturbances, were less frequent among patients receiving the VA regimen.

Furthermore, patients who experienced treatment failure with PAC and were subsequently switched to VA achieved successful eradication, reinforcing the potential of VA dual therapy. In contrast, only one-third of those switched from VA to PAC responded, highlighting the limitations of PAC therapy, particularly in the context of increasing clarithromycin resistance. These findings align with existing evidence from East Asia, where vonoprazan-based regimens have demonstrated high eradication rates [7]. Randomized controlled trials, including those by Kiyotoki et al 2020 [15] and Murakami et al 2016 [11], reported eradication rates between 90% and 95%. In addition, the real-world study has also further validated the effectiveness of vonoprazan-based therapies in clinical practice [17].

The second-line switching findings are clinically relevant, as all PAC-to-VA switchers achieved eradication; however, these results should be considered exploratory because of the small number of patients.

Our findings are also in alignment with the retrospective study by Gao et al 2025, which demonstrated a 95.6% eradication rate with VA dual therapy in elderly Chinese patients, along with a low AE incidence of 9.5% and high adherence (98.7%) [18]. These comparable outcomes suggest that VA dual therapy maintains its effectiveness and safety across varied populations and healthcare systems. The high tolerability and simplified dosing schedule of VA therapy may contribute to better patient compliance and treatment success, especially in real-world practice.

A critical concern in the management of *H. pylori* infection in India is the increasing rate of antibiotic resistance, particularly to clarithromycin, which significantly diminishes the efficacy of traditional PPI-based triple regimens such as PAC. Indian studies have documented high resistance rates to commonly used antibiotics such as metronidazole, clarithromycin, and levofloxacin, highlighting the pressing need for more effective alternative treatment strategies [19]. In this context, vonoprazan, a novel P-CAB, demonstrates several pharmacological advantages over conventional PPIs. Unlike PPIs, which require acid activation and exhibit delayed onset, vonoprazan acts rapidly and inhibits gastric hydrogen/potassium adenosine triphosphatase (H^+^/K^+^-ATPase) enzyme in a K^+^-competitive and reversible manner, achieving potent and sustained acid suppression from the first dose [11]. This enhanced acid control creates an optimal intragastric environment for antibiotics, improving their stability and antimicrobial efficacy. Moreover, the metabolism of vonoprazan is not affected by cytochrome P450 2C19 (CYP2C19) genetic polymorphisms, which often compromise the performance of PPIs in diverse populations [20]. Both randomized controlled trials and real-world studies from East Asia have consistently reported superior eradication rates with vonoprazan-based therapies, particularly in settings where clarithromycin resistance is common; however, resistance testing was not performed in this study [11, 21]. These attributes make vonoprazan-based dual therapy a compelling and potentially more effective alternative for *H. pylori* eradication, especially in regions like India, where rising antimicrobial resistance poses a substantial therapeutic challenge.

The faster complete symptom resolution observed with the VA regimen in our study aligned with previous studies showing significantly faster improvement in erosive esophagitis symptoms with vonoprazan as compared to PPI lansoprazole in patients with acid-related disorders due to its rapid onset and stronger acid inhibition [22]. Similarly, in refractory gastroesophageal reflux disease (GERD) patients, vonoprazan showed a more potent gastric suppression and better improvement in symptoms than PPI [23]. Recent evidence supports the role of P-CABs in *H. pylori* eradication. Kanu et al reported higher eradication rates with vonoprazan-based triple therapy than conventional PPI-based triple therapy. Although our study assessed VA dual therapy, its findings are broadly consistent with the wider P-CAB literature [24].

The cost-effectiveness analysis of our study showed that the VA regimen was associated with lower median treatment costs and a more favorable CER compared with the PAC regimen. PAC regimen costs INR 2,360 more per patient (USD 28.43) and is about 3.31 times more expensive than the VA regimen. These findings are in line with emerging evidence, which reported that vonoprazan-based therapies reduced total medical expenditure by achieving higher eradication rates and minimizing the need for retreatment [25, 26]. Additionally, the recent equivalence in pricing between vonoprazan and standard PPIs within the Indian market further supports the cost-effectiveness of the VA regimen as a first-line treatment approach [27, 28].

This study offers several strengths. The real-world, pragmatic design enhances the clinical applicability of the findings, reflecting routine prescribing behaviors and treatment responses. Additionally, the inclusion of second-line treatment outcomes offers valuable insights into sequencing strategies following treatment failure.

This study has certain limitations. The single-center design may restrict the generalizability of the findings, and the observational nature of the study limits the ability to draw definitive causal conclusions. Furthermore, this study lacks sufficient power to detect statistically significant differences in the primary outcome, which may limit the robustness of the results. Therefore, the present findings cannot directly establish VA effectiveness in confirmed clarithromycin-resistant *H. pylori* infection. No multivariable adjustment for baseline variables was performed because of the small sample size and limited number of outcome events. Future large-scale prospective studies integrating local resistance data would strengthen the understanding of treatment efficacy and inform tailored therapeutic approaches. Another limitation is the use of repeated RUT as the eradication endpoint. Although practical in this retrospective real-world setting, RUT may be affected by biopsy site, sampling adequacy, patchy *H. pylori* colonization, and recent acid-suppressive, antibiotic, or bismuth use. Therefore, eradication should be interpreted as RUT-confirmed, and future prospective studies should preferably use urea breath or stool antigen testing. The economic findings should be interpreted cautiously, as this was a basic cost comparison with a simple cost-per-eradication estimate, not a formal cost-effectiveness analysis. No sensitivity analyses, quality-adjusted outcome assessments, or incremental cost-effectiveness analyses were performed. The retrospective, non-randomized, single-center design may introduce selection bias, confounding, and information bias. In addition, the small sample size, some missing data, and lack of formal sample-size calculation may limit statistical power and generalizability. Restricted mean survival time analysis was not performed, which may be considered in future prospective studies.

The evidence from this study suggests that VA dual therapy appears to be a promising first-line option in Indian clinical settings, particularly where clarithromycin resistance is suspected, although resistance status was not microbiologically conformed. Its safety profile makes it a practical alternative to conventional triple therapy, especially for patients with previous treatment failure or intolerance to multi-drug regimens. The proportional hazards assumption was violated in the Cox model; therefore, the HR estimates should be interpreted cautiously, with greater emphasis on the Kaplan–Meier and median time-to-resolution findings. As treatment allocation was not randomized, unmeasured clinical factors influencing physician choice of VA or PAC may have introduced residual confounding. Also, the formal adherence or compliance data were not consistently documented in the medical records; therefore, treatment adherence could not be systematically assessed in this retrospective study. Local antimicrobial resistance patterns from the study center were not available.

This study paves the way for future research to build upon these findings; large-scale, multi-center randomized controlled trials are essential to validate the comparative efficacy of VA versus PAC and other regimens in diverse Indian populations. Including regular antibiotic resistance testing in both clinical practice and research will help improve treatment choices and update national guidelines for managing *H. pylori.*

### Conclusions

This real-world, observational study indicates that vonoprazan-based dual therapy is a promising and practical alternative to traditional PPI-based triple therapy for *H. pylori* eradication in Indian clinical settings. With numerically higher eradication rates and better tolerability both as a first-line and rescue regimen, VA dual therapy may be a promising and practical alternative to traditional PPI-based triple therapy for *H. pylori* eradication in Indian clinical settings, particularly where clarithromycin resistance is suspected; however, confirmation in larger prospective studies with susceptibility testing is required. Its favorable safety profile, ease of administration, and cost-effectiveness further support its use, especially among those with prior treatment failure or intolerance to multi-drug regimens.

## Data Availability

The datasets generated and/or analyzed during the current study are available from the corresponding author on reasonable request.

## Abbreviations

*H. pylori*: *Helicobacter pylori*
VA: vonoprazan–amoxicillin
PAC: proton pump inhibitor–amoxicillin–clarithromycin
PPI: proton pump inhibitor
P-CAB: potassium-competitive acid blocker
RUT: rapid urease test
ADR(s): adverse drug reaction(s)
AE(s): adverse event(s)
CTCAE: Common Terminology Criteria for Adverse Events
BID: twice daily (*bis in die*)
TID: three times daily (*ter in die*)
QID: four times daily (*quater in die*)
ALT: alanine aminotransferase
AST: aspartate aminotransferase
ULN: upper limit of normal
HIV: human immunodeficiency virus
CD4: cluster of differentiation 4
INR: Indian rupee
USD: US dollar
IQR: interquartile range
SD: standard deviation
HR: hazard ratio
CI: confidence interval
CER: cost-effectiveness ratio
ΔC: cost difference
GERD: gastroesophageal reflux disease
CYP2C19: cytochrome P450 2C19
H^+^/K^+^-ATPase: hydrogen/potassium adenosine triphosphatase
MALT: mucosa-associated lymphoid tissue
